# Sounds of danger and post-traumatic stress responses in wild rodents: ecological validity of a translational model of post-traumatic stress disorder

**DOI:** 10.1038/s41380-023-02240-7

**Published:** 2023-09-06

**Authors:** Hagit Cohen, Michael A. Matar, Doron Todder, Carmit Cohen, Joseph Zohar, Hadas Hawlena, Zvika Abramsky

**Affiliations:** 1 Faculty of Health Sciences, Ben-Gurion University of the Negev, Israel & Ministry of Health, Anxiety and Stress Research Unit, Beer-Sheva Mental Health Center, Beer-Sheva, Israel; 2 Department of Psychology, Ben-Gurion University of the Negev, Beer-Sheva, Israel; 3 Post-Trauma Center, Sheba Medical Center, Tel Aviv University, Tel Aviv, 52621 Israel; 4 Mitrani Department of Desert Ecology, The Jacob Blaustein Institutes for Desert Research, Ben-Gurion University of the Negev, Midreshet Ben-Gurion Israel, Sde Boker, 8499000 Israel; 5 Department of Life Sciences and Ramon Science Center, Ben-Gurion University of the Negev, Beer-Sheva, 84105 Israel

**Keywords:** Neuroscience, Psychiatric disorders

## Abstract

In the wild, animals face a highly variable world full of predators. Most predator attacks are unsuccessful, and the prey survives. According to the conventional perspective, the fear responses elicited by predators are acute and transient in nature. However, the long-term, non-lethal effects of predator exposure on prey behavioral stress sequelae, such as anxiety and post-traumatic symptoms, remain poorly understood. Most experiments on animal models of anxiety-related behavior or post-traumatic stress disorder have been carried out using commercial strains of rats and mice. A fundamental question is whether laboratory rodents appropriately express the behavioral responses of wild species in their natural environment; in other words, whether behavioral responses to stress observed in the laboratory can be generalized to natural behavior. To further elucidate the relative contributions of the natural selection pressures influences, this study investigated the bio-behavioral and morphological effects of auditory predator cues (owl territorial calls) in males and females of three wild rodent species in a laboratory set-up: *Acomys cahirinus*; *Gerbillus henleyi*; and *Gerbillus gerbillus*. Our results indicate that owl territorial calls elicited not only “fight or flight” behavioral responses but caused PTSD-like behavioral responses in wild rodents that have never encountered owls in nature and could cause, in some individuals, enduring physiological and morphological responses that parallel those seen in laboratory rodents or traumatized people. In all rodent species, the PTSD phenotype was characterized by a blunting of fecal cortisol metabolite response early after exposure and by a lower hypothalamic orexin-A level and lower total dendritic length and number in the dentate gyrus granule cells eight days after predator exposure. Phenotypically, this refers to a significant functional impairment that could affect reproduction and survival and thus fitness and population dynamics.

## Introduction

Post-traumatic stress disorder (PTSD) is a potentially chronic impairing disorder involving cognitive, emotional, and physiological failure to adequately process and/or recover from exposure to a traumatic experience [1]. The memories of a traumatic event remain vivid, chronically active, and disruptive over long periods of time, together with the emotions at the time of the event, shaping symptoms such as intrusive thoughts, physiological hyperarousal, active avoidance of traumatic reminders, and negative alterations in cognitions and mood.

From an evolutionary-ecological perspective, memory is distinctly advantageous for survival. An organism’s ability to form and retain a record, especially of threatening events, and to accumulate and maintain this information to allow for ready access and ongoing updating, that is, to form memories, confers the ability to anticipate danger and prepare for or avoid it. In natural habitats, animals are required to continually assess and evaluate the potential risks they face while balancing the need for food acquisition with the need for safety, considering the dynamic changes in resource availability, competition intensity, and predation risk. Therefore, animals have evolved a variety of mechanisms for evaluating the potential risk of predation, implementing antipredator decision-making strategies, and developing adaptive behavioral responses to these conditions [2–7]. For example, when faced with predation risk, some prey individuals may alter their selection of micro or macro-habitats, seeking concealment, while others may exhibit morphological changes such as alteration of coloration, to enhance camouflage and increase the trade-off between food acquisition and safety [6]. According to the conventional perspective, the fear responses elicited by predators are acute and transient in nature [8]. The prey identifies the predator and initiates a behavioral response (fleeing, fighting, or freezing). Following the encounter, the animal either survives or dies. If survival occurs, it is commonly assumed that the animal resumes its normal activities [9] and physiological equilibrium is restored. However, studies on commercial strains of rats and mice have shown that non-lethal, long-lasting effects of predators, such as fear, anxiety, or post-traumatic responses, can have a significant impact on individual morphology, behavior, and reproductive success [10–14]. A fundamental question is thus the extent to which experimental findings using a commercial strain of rats or mice can be generalized to real-world situations. In particular, it is not known if there are “post-trauma” symptoms for animals in the wild or which behavioral stress responses observed in the laboratory appear in wild animals in the field [9, 15]. Should we expect that in natural communities the non-lethal enduring effects of predators will be significant as well, and whether these effects will have a greater impact on wild prey populations (and on predators) in terms of individual behavior, population dynamics, and reproductive success compared to the non-lethal short-term effects and the lethal effects.

To fill the above knowledge gaps, we investigated the effects of predator stress on the behavioral, molecular, and morphological responses of wild rodent species in a laboratory setup. We studied males and females of three rodent species: *Acomys Cahirinus* (*A-Cahirinus*); *Gerbillus Henleyi (G-Henleyi)*; and *Gerbillus Gerbillus (G-Gerbillus)* (Supplement #1).

Gerbils are particularly interesting for the study of PTSD as their adrenal glands are comparable to the adrenals of the humans, due to their high concentration of ascorbic acid and their secretion of cortisol, unlike most rodents [16–18].

The goal of this study was to evaluate the bio-behavioral and morphological effects of predator cues (playback of tape-recorded owl calls) in males and females of three wild rodent species. To this end, we used (a) the cut-off behavioral criteria (CBC) classification model. In this model, populations of exposed rodents are classified based on the degree of their individual behavioral response, creating three distinct groups: “extreme,” “partial,” or “minimal” behavioral response (EBR, PBR, and MBR, respectively) (Supplement #2), (b) measured fecal cortisol metabolite (FCM) levels 1–2 h after predator stress, (c) examined orexin-A levels in the hypothalamus eight days after exposure, and (d) we evaluated changes in the cytoarchitecture of the hippocampus using the Golgi-Cox method.

## Materials and methods

### Animals

We used two gerbilline species (*G-Henleyi* and *G-Gerbillus*) and one murine species (*A-Cahirinus*), which are burrow dwellers of the Negev Desert in Israel. Twenty-three *G-Henleyi* (10 females and 13 males), fifteen *G-Gerbillus* (8 females and 7 males), and thirty-five *A-Cahirinus* (19 females and 16 males) specimens were used in this study.

All rodents were descendants of wild rodents captured in the vicinity of the Negev Desert in Southern Israel. After trapping, the animals were transported to Midreshet Ben-Gurion, where they were housed in pairs (male and female) and bred for 6–8 generations. Considering the small number of generations compared to the evolution of the species themselves [19, 20], these rodents, which were kept and bred under semi-natural conditions, can be considered wild rather than domesticated [20].

All rodents used in the experiments were adults and were acclimatized to the conditions to which they were later exposed for at least one month prior to the habituation experiments. Rodents were housed in pairs in plastic cages (60 × 50 × 40 cm) under controlled temperature (25 ± 1 °C) and humidity (30 ± 5%) conditions, with a photoperiod of 12D:12 L (lights on at 0700 h) and with sawdust and dried grass as bedding material. They were provided daily with millet seeds ad libitum and fresh alfalfa (*Medicago* sp.) as a water source following Hawlena et al. [21]. All experimental procedures were conducted between 13:00–16:00.

### Experimental design

The base level of anxiety-related behavior (and the intensity of the response to stressor) were determined using the open field test (OFT). This test was chosen over the elevated plus maze (EPM) because repeated testing on the same animals reduces its validity [22]. After being exposed to predator cues for 10 min, fresh feces were collected to test for cortisol levels. Behavioral tests were conducted using the OFT, EPM, and ASR tests on day 7. Exploratory behavior in the EPM serves as the main platform for the assessment of overall behavior, and the ASR paradigm provides a precise quantification of hyperalertness in terms of the magnitude of response and habituation to the stimulus. These data were used to classify the animals into behavioral response groups. The rats were then sacrificed, and brains were collected for morphology analysis. The baseline FCM levels were evaluated in *G-Gerbillus* specimens.

### Stress exposure

Individual rodents were exposed to tape-recorded territorial calls of owls. Stress was induced by placing the test animals in a plastic cage (40 × 40 × 40 cm) that was situated on a yard paving stone for 10 min in a closed environment. Sound was transmitted into the arena using a small loudspeaker (2.5 inch, 10 Ω, 0.2 W max) placed on the rear wall, 45 cm above the floor, and connected to a cassette recorder. The sound levels of the tape-recorded owl calls were standardized to approximately 60–70 dB. After 10 min of playback of the owl calls, we turned off the recording device and left the rodents in the arena for another 5 min of silence. This period simulates the hunting pattern of the owl, which, following a period of territorial calling, flies to a hunting perch where it waits in complete silence to pounce on its prey [23].

### Behavioral assessments

All behavioral tests were video-recorded using the ETHO-VISION program (Noldus), by an investigator blinded to the experimental protocol. Behaviors of specimens were assessed using OFT, EPM and ASR, as described previously [24–26]. Detailed protocols are described in Supplementary Information #3:1.

### Fecal cortisol metabolites

This non-invasive technique for measuring steroid metabolites in fecal samples has been established in an increasing number of species [27–33]. Fresh fecal samples were collected, and any feces contaminated with urine was excluded from the analysis. The fecal samples were placed in 2 mL microfuge tubes and immediately frozen at −20 °C for subsequent analysis. Hormones were extracted using the method described by Gutman et al. [34]. Fecal cortisol metabolites were measured using a radioimmunoassay kit (ICN Biomedical, Inc.) in duplicate.

### Brains

24-hours after the behavioral tests, between 12:00 and 14:00, the animals were deeply anesthetized and perfused intracardially with saline. The brains were immediately removed, and the hypothalamus was dissected. Each hypothalamus was washed in saline, weighed, and frozen at −80 °C until later use. Brains (without hypothalamus) were prepared using a rapid Golgi kit (FD Neurotechnologies, USA) according to the manufacturer’s instructions (Supplementary Information 3:2).

### Orexin-A levels in the hypothalamus

The hypothalamus samples were homogenized (PRO Scientific Inc., CT, USA) at 24,000 rpm for 2 min in ice-cold PBS and centrifuged at 15,000 rpm for 30 min at 4 °C. The supernatant was separated and stored at −80 °C. Orexin-A levels in brain tissue were measured using a commercially available enzyme-linked immunoassay kit (MyBioSource, Inc., San Diego, CA, USA) according to the manufacturer’s protocol, in duplicates.

### Statistical analyses

All data are expressed as mean±standard error of the mean (SEM), and statistical analyses were performed using a two-way analysis of variance (ANOVA). For the OFT, statistical analyses were performed using repeated measures (RM)-ANOVA. Where significant group effects were detected, the Bonferroni test assessed significant post-hoc differences between groups. The prevalence of PTSD-classified groups was tested in relation to rodent species group or sex using cross-tabulation, chi-square tests, and logistic regression analysis. To gain an additional understanding of the relationship between behavioral and molecular/morphology measures, Pearson’s correlation analysis was performed.

## Results

To preclude the possible effects of basal pre-trauma anxiety, which can be a risk factor for the development and persistence of PTSD, animals were first evaluated in the OFT under basal conditions. We found that the level of anxiety-related behavior was low: the path length or thigmotaxis all indicated a low level of anxiety-related behavior without any differences among the species or between sexes. In all species, exposure to the owl cues reduced the percentage of time spent in the inner and middle zones and increased the time spent in the outer zone, which are common indices of anxiety-related behavior (Supplementary Information #4:0).

**Fig. 1:**
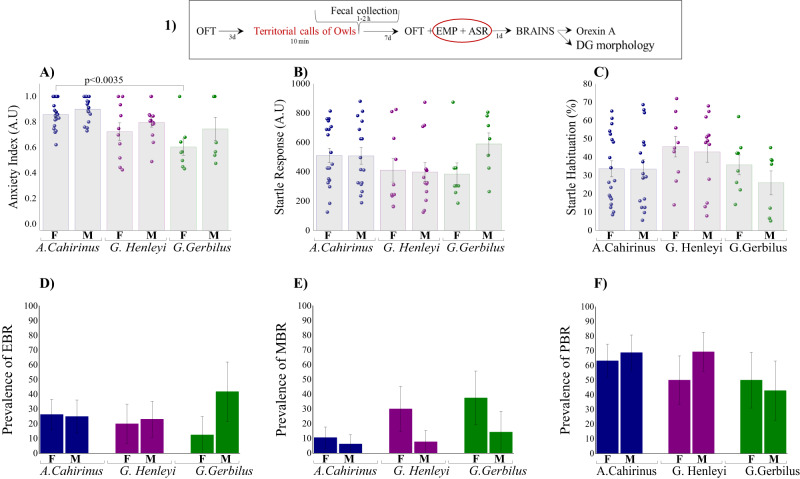
The long-term effects of predator cue exposure on behavior. The top panel (1) depicts the experimental protocol. The red circle signifies the behavioral test performed, for which the results are shown. **A** Anxiety Index which integrates the measured EPM behavioral measures. **B** Startle amplitude in the acoustic startle response (ASR) paradigm. **C** Percentage of startle habituation in the ASR paradigm. **D** Prevalence of extreme behavioral response (EBR) (in percentages), **E** Prevalence of minimal behavioral response (MBR) (in percentages), **F** Prevalence of partial behavioral response (PBR) (in percentages). Owl territory calls had long-lasting influences on rodent behavior; all species reacted significantly to predator cue stress in terms of anxiety-related behavior in the EPM and ASR paradigms 7 days after exposure. In addition, predator cue exposure causes post-traumatic stress disorder (PTSD)-like behavioral responses in wild rodents that have never encountered owls. Data are presented as data points and mean ± SEM and percentage.

In terms of anxiety index (Fig. 1A), two-way ANOVA revealed a significant effect of species (F(2,67) = 10.2, *p* < 0.00015, respectively) and sex (F(2,67) = 4.9, *p* < 0.035, respectively). Bonferroni test confirmed that female *A-Cahirinus* exposed to predator stress spent significantly less time in the open arms, entered the open arms less frequently, and exhibited a higher anxiety index than female *G-Gerbillus* (*p* < 0.005, *p* < 00002, and *p* < 0.0035, respectively) (Supplementary Information #4:1 for all EPM parameters).

No species or sex differences in the ASR or startle habituation (Fig. 1B, C) were observed.

### Relative prevalence rates according to CBC-classification

The species didn’t differ in their overall responses to imposed predator cue. There were no significant differences in the prevalence of EBR (PTSD phenotype) (Fig. 1D), MBR (Fig. 1E), or PBR (Fig. 1F) among species or between sexes. No predictor variable reached significance.

The prevalence of EBR among *A-Cahirinus* females was **26.3%** (5/19), and **25.0%** (4/16) among *A-Cahirinus* males. Moreover, in *A-Cahirinus*, 2 females (10.5%) and 1 male (6.25%) fulfilled the criteria for MBR, and 12 females (63.15%) and 11 males (68.75%) were classified as PBR. In *G-Henleyi*, the prevalence of EBR in females was **20.0%** (2/10) and **23.0%** (3/13) among males. Moreover, 3 females (30.0%) and 1 male (7.69%) fulfilled the criteria for MBR, and 5 females (50.0%) and 9 males (69.23%) were classified as PBR. In *G-Gerbillus*, the prevalence of EBR females was **12.5%** (1/8) and **42.85%** (3/7) among males, without significant differences, likely due to the small sample size. Moreover, 3 females (37.5%) and 1 male (14.3%) fulfilled the criteria for MBR, and 4 females (50.0%) and 3 males (37.5%) were classified as PBR. In *G-Gerbillus*, although a χ2 analysis indicated that sex did not affect the prevalence of the extremes in the behavioral response to stress (Fisher exact *p* = 0.023, not significant), marked behavioral differences were evident between female and male rodents. Male *G-Gerbillus* showed a higher prevalence of EBR than female rats.

Overall, the prevalence of EBR in *A-Cahirinus* species was 25.71% (9/35), while in *G-Henleyi* or *G-Henleyi* species, it was 21.7% (5/23) and 26.67% (4/15), respectively.

### FCM

In *A-Cahirinus* and *G-Henleyi*, measurements were made only after exposure to stress, that is, without basal level measurements. Overall, two-way ANOVA revealed a significant effect of species on FCM levels (F(1,67) = 6.85, *p* < 0.015).

**Fig. 2:**
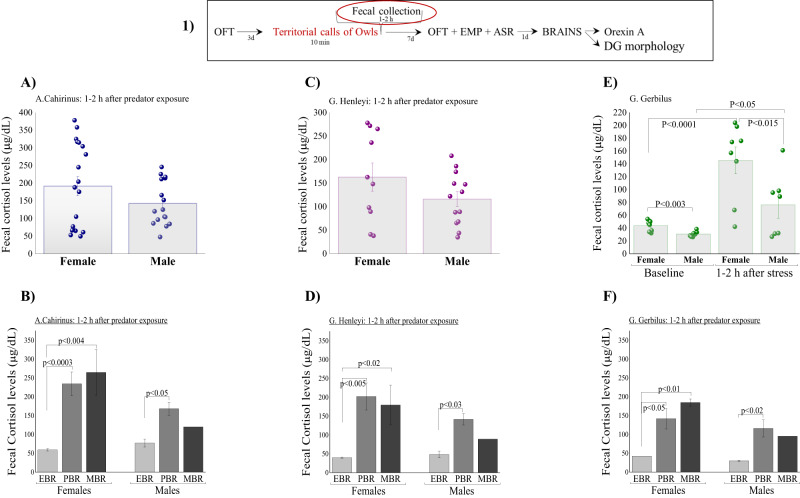
The effects of predator cue exposure on fecal cortisol metabolite (FCM) levels. The top panel (1) depicts the experimental protocol. The red circle signifies the behavioral test performed, for which the results are shown. **A** The FCM levels, collected 1–2 h after predator stress exposure, in female and male *Acomys cahirinus*. **B** The FCM levels in *Acomys cahirinus* according to the affected groups (classified using the cut-off behavioral criteria (CBC) method). **C** The FCM levels, collected 1–2 h after predator stress exposure, in female and male *Gerbillus henleyi*. **D** The FCM levels in *Gerbillus henleyi* according to the affected groups (classified using the CBC method). **E** The FCM levels, collected 1–2 h after predator stress exposure, in female and male *Gerbillus gerbillus*. **F** The FCM levels in *Gerbillus gerbillus* according to the affected groups (classified using the CBC method). Although we tested basal FCM concentrations in G. gerbillus before predator exposure and found that the FCM concentrations were relatively low and significantly elevated in response to the stressor compared to their baseline levels, it is still unclear whether the poor FCM response to predator stress and the dysregulation observed in the acute aftermath of trauma represent an existing pre-trauma vulnerability trait or develops from the exposure to the trauma itself. Data are presented as data points and mean ± SEM.

In *A-Cahirinus*, FCM concentrations were not homogeneous and wide scattering was detected (Fig. 2A). No significant differences were found in FCM concentrations between females and males, but there was a trend of lower FCM concentrations in males (F(1,29) = 2.9, *p* = 0.09). Looking at FCM concentrations between PTSD-classified groups (Fig. 2B), two-way ANOVA revealed a significant difference in FCM concentrations between groups (F(2,29) = 9.7, *p* < 0.0006). In females, Bonferroni post-hoc tests confirmed significantly lower FCM concentrations in EBR than in PBR (*p* < 0.0003) and MBR (*p* < 0.004) rodents. In males, Bonferroni tests confirmed significantly lower FCM concentrations in EBR than in PBR (*p* < 0.05) rodents.

In *G-Henleyi*, no significant differences were found in FCM levels between females and males (Fig. 2C). Two-way ANOVA revealed a significant effect of groups (F(2,17) = 8.3, *p* < 0.0035) on FCM concentrations (Fig. 2D). In females, Bonferroni tests confirmed significantly lower FCM concentrations in EBR than in PBR (*p* < 0.005) and MBR (*p* < 0.05) rodents. In males, Bonferroni post-hoc tests confirmed significantly lower FCM concentrations in EBR than in PBR (*p* < 0.03).

In *G-Gerbillus*, we first monitored baseline FCM concentrations and subsequently measured the post-stress FCM response. RM-ANOVA revealed a significant effect of sex (F(1,16) = 8.3, *p* < 0.015) and stress (F(1,16) = 26.2, *p* < 0.00025) (Fig. 2E). In both females and males, stress significantly increased FCM concentration relative to the baseline conditions. Bonferroni tests indicated that all exposed rodents exhibited significantly elevated mean FCM concentrations compared with those in the control group (females: *p* < 0.0001 and males: *p* < 0.015). Moreover, both under baseline conditions and stress conditions the FCM concentrations were significantly higher in females than in males (*p* < 0.003 and *p* < 0.05, respectively). Two-way ANOVA revealed a significant effect of groups (F(2,9) = 7.76, *p* < 0.015) on FCM concentrations (Fig. 2F). No effects were observed for sex, but there was a trend of lower FCM concentrations in males (F(1,9) = 3.6, *p* = 0.09). In females, Bonferroni tests confirmed significantly lower FCM concentrations in EBR than in PBR (*p* < 0.05) and MBR (*p* < 0.01). In males, Bonferroni tests confirmed significantly lower FCM concentrations in EBR than in PBR (*p* < 0.02).

We conducted regression analyses to further understand the relationship between FCM levels and behavioral measures, regardless of CBC classification (Table 1). *In A-Cahirinus, G-Henleyi and G-Gerbillus* Pearson’s correlation analysis revealed that FCM concentrations were significantly and negatively correlated with the anxiety index.

**Table 1:** Correlation analysis between anxiety index and fecal cortisol levels.

	Anxiety index		
	*Acomys Cahirinus*	*Gerbillus Henleyi*	*Gerbillus Gerbillus*
Fecal cortisol levels (µg/dL)	*r* = −0.57, *p* < 0.0004, *n* = 35	*r* = −0.42, *p* < 0.05, *n* = 23	*r* = −0.8, *p* < 0.0004, *n* = 15
Orexin-A levels (pg/ml)	*r* = −0.53, *p* < 0.001, *n* = 35	*r* = −0.61, *p* < 0.0025, *n* = 23	*r* = −0.81, *p* < 0.0003, *n* = 23
DG dendritic Number	*r* = −0.68, *p* < 0.0025, *n* = 24	*r* = −0.61, *p* < 0.003, *n* = 23	*r* = −0.89, *p* < 0.0001, *n* = 15
DG dendritic Length (µm)	*r* = −0.57, *p* < 0.004, *n* = 24	*r* = −0.55, *p* < 0.007, *n* = 23	*r* = −0.75, *p* < 0.002, *n* = 15

Marked correlations are significant at *p* < 0.05.

### Brain orexin-A levels

**Fig. 3:**
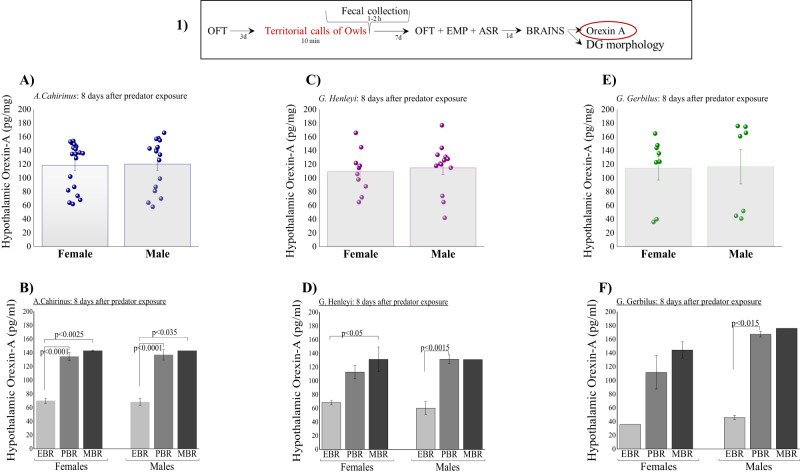
The long-term effects of predator cue exposure on hypothalamic levels of orexin A. (1) The top panel depicts the experimental protocol. The red circle signifies the test performed whose results are shown. **A** Hypothalamic levels of orexin-A (ORX-A) in females and males *Acomys cahirinus* species. **B** Hypothalamic levels of ORX-A in *Acomys cahirinus* species according to the affected groups (according to the cut-off behavioral criteria (CBC) method). **C** Hypothalamic levels of ORX-A in females and males *Gerbillus henleyi* species. **D** Hypothalamic levels of ORX-A in *Gerbillus henleyi* species according to the affected groups (according to the CBC method). **E** Hypothalamic levels of ORX-A in females and males *Gerbillus gerbillus* species. **F** Hypothalamic levels of ORX-A in *Gerbillus gerbillus* species according to the affected groups (according to the CBC method). Data are presented as data points and mean ± SEM.

Changes in total orexin-A levels in rodent brains following stress exposure are shown in Fig. 3. Overall, no significant differences were found in orexin-A levels among species or between sexes.

In *A-Cahirinus*, no significant differences were found in orexin-A levels between females and males (Fig. 3A). Two-way ANOVA revealed a significant effect of groups (F(2,29) = 38.2, *p* < 0.00016) (Fig. 3B). In both sexes, Bonferroni tests confirmed significantly lower orexin-A levels in EBR than in PBR (*p* < 0.0001 for both females and males) and MBR (females: *p* < 0.0025 and males: *p* < 0.035).

In *G-Henleyi*, no significant differences were found in orexin-A levels between females and males (Fig. 3C). Two-way ANOVA revealed a significant effect of groups (F(2,17) = 15.2, *p* < 0.0002) (Fig. 3D). In females, Bonferroni tests confirmed significantly lower orexin-A levels in EBR than in MBR (*p* < 0.05). In males, Bonferroni post-hoc tests confirmed significantly lower orexin-A levels in EBR than in PBR (*p* < 0.0015).

In *G-Gerbillus*, no significant differences were found in orexin-A levels between females and males (Fig. 3E). Two-way ANOVA revealed a significant effect of groups (F(2,9) = 14.4, *p* < 0.002) on orexin-A levels (Fig. 3F). In males, Bonferroni tests confirmed significantly lower orexin-A levels in EBR than in PBR (*p* < 0.015).

*In A-Cahirinus*, *G-Henleyi and Gerbillus-G* Pearson’s correlation analysis revealed that orexin-A levels were significantly and negatively correlated with the anxiety index (Table 1).

### Morphology of DG granular neurons

**Fig. 4:**
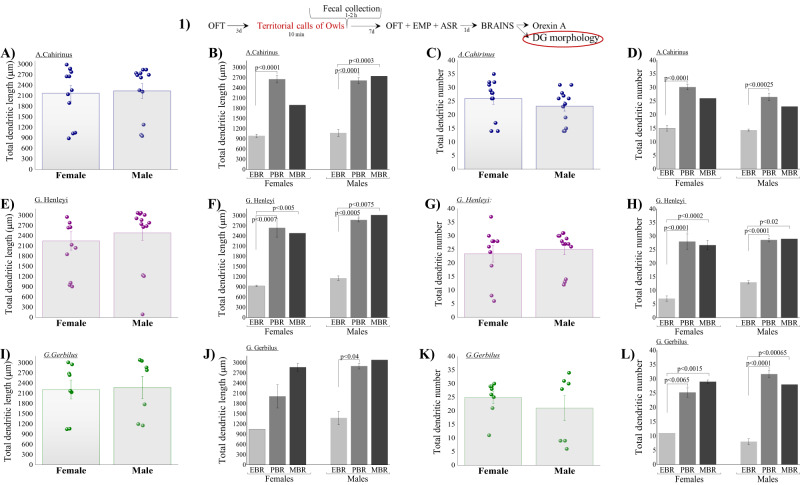
The long-term effects of predator cue exposure on morphology of dentate gyrus (DG) granular neurons. (1) The top panel depicts the experimental protocol. The red circle signifies the test performed, for which the results are shown. **A** Quantitative analysis of total dendritic length (μm), and (**C**) total dendritic number of dentate gyrus granule cells from the suprapyramidal blade in female and male *Acomys cahirinus*, *Gerbillus henleyi* (**E**, **G**, respectively), and *Gerbillus gerbillus* (**I**, **K**, respectively). **B** Quantitative analysis of total dendritic length (μm), and (**D**) total dendritic number of dentate gyrus granule cells according to the affected groups in *Acomys cahirinus*, *Gerbillus henleyi* (**F**, **H** respectively), and *Gerbillus gerbillus* (**J**, **L**, respectively). Data are presented as data points and mean ± SEM.

**Fig. 5:**
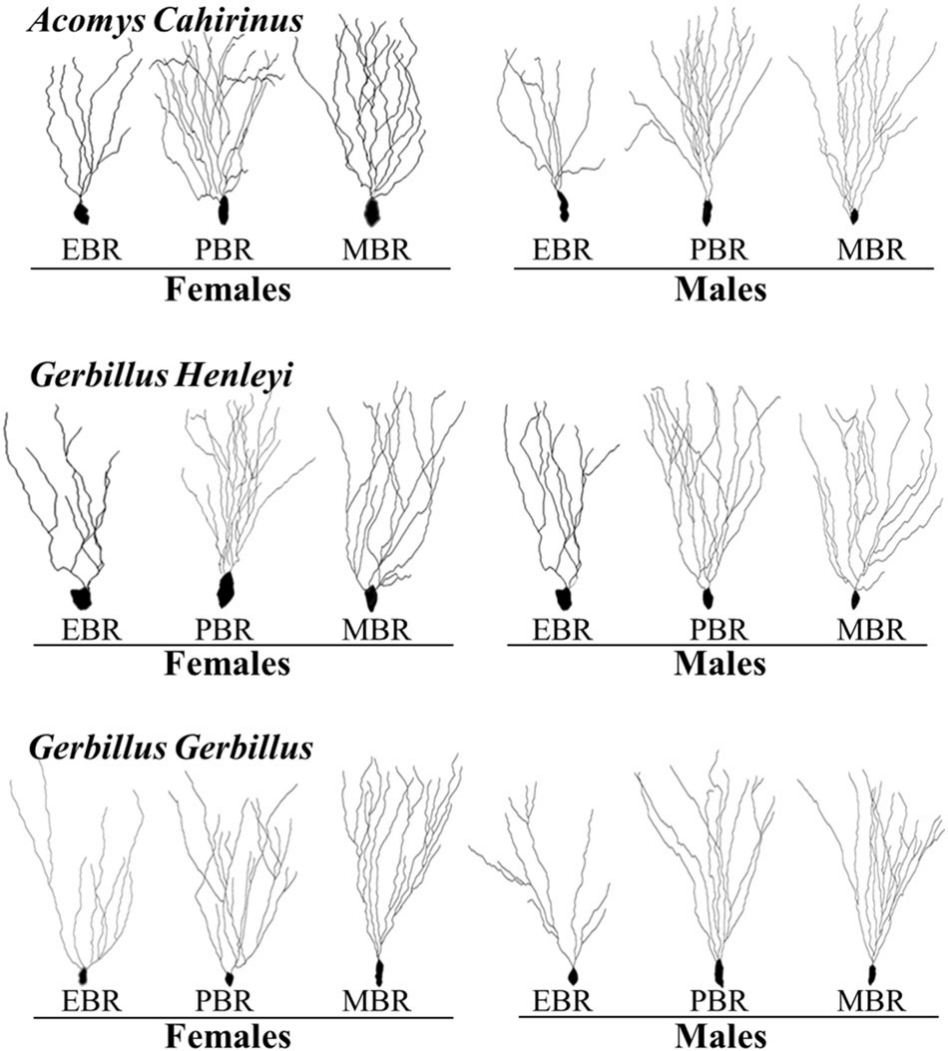
Computer-generated plots of reconstructions of the dendritic trees from granule cells of female and male *Acomys cahirinus*, *Gerbillus henleyi*, and *Gerbillus gerbillus*.

Overall, no significant differences were found in the total dendritic length or number among species or between sexes (Figs. 4–5).

In *A-Cahirinus*, no significant differences were found in the total dendritic length (Fig. 4A) or number (Fig. 4C) between females and males. Eight days after predator stress, the total dendritic length (Fig. 4B) and total dendritic number (Fig. 4D) were significantly lower in both female and male EBR animals than in the PBR group (*p* < 0.0001 and *p* < 0.00025, respectively).

In *G-Henleyi*, no significant differences were found in the total dendritic length (Fig. 4E) or number (Fig. 4G) between females and males. The total dendritic length (Fig. 4F) and the total dendritic number (Fig. 4H) were significantly lower in both female and male EBR animals than in the PBR (*p* < 0.0001 and *p* < 0.0002, respectively) and MBR (*p* < 0.0001 and *p* < 0.02, respectively) groups.

In *G-Gerbillus*, no significant differences were found in the total dendritic length (Fig. 4I) or number (Fig. 4K) between females and males. The total dendritic length (Fig. 4J) and the total dendritic number (Fig. 4L) were significantly lower in both female and male EBR animals than in the PBR (*p* < 0.0065 and *p* < 0.00015, respectively) and MBR (*p* < 0.0001 and *p* < 0.00065, respectively) groups.

*In A-Cahirinus*, *G-Henleyi and Gerbillus-G* Pearson’s correlation analysis revealed that dendritic number and length were significantly and negatively correlated with the anxiety index (Table 1).

## Discussion

This study aimed to explore the application of our standard model and procedures for translational studies of PTSD in laboratory rodents to three groups of wild rodents of both sexes, specifically *A-Cahirinus*, *G-Henleyi*, and *G-Gerbillus*, in an adapted format. This study employed recordings of owl territorial calls as the trigger, to which they had never previously been exposed in their lifetime. The results indicate that this evolutionary trauma cue indeed elicited not only the acute “fight or flight” responses to the potential predator threat, but also significant long-term behavioral, neurobiological, and morphological sequelae that are completely in line with our previous findings in laboratory rodents. The results similarly mirror findings from clinical studies in traumatized patients, changes that could significantly compromise functions related to survival and reproduction.

All species reacted to the playback of owl vocalizations significantly in terms of anxiety-related behavior in the EPM, OFT, and ASR paradigms and in the overall pattern of resultant FCM concentrations, orexin-A levels, and DG dendritic arborization, validating the potentially traumatizing effect of the stressor. However, bio-behavioral stress responses showed extensive individual phenotypic heterogeneity at the baseline and after predator exposure within the species. Marked behavioral (i.e., phenotypic) differences were evident in all paradigms among individuals within each species after stress exposure.

Separating out the more clearly affected animals using CBC enabled a more precise assessment of the magnitude and character of the predator effect [25]. Accordingly, the prevalence of severely behaviorally affected animals (PTSD phenotype) in *A-Cahirinus* was 26.3% in females and 25% in males, whereas in *G-Henleyi* it was 20% in females and 23% in males. The prevalence of the PTSD phenotype in *G-Gerbillus* was 12.5% in females and 41.85% in males. Nevertheless, there were no statistically significant differences in the prevalence rates of PTSD phenotype among species or between sexes, but it is possible that the absence of species and sex differences is a result of the low statistical power due to the limited sample size. Future studies should use larger sample sizes. Taken together, predator cue exposure induces PTSD-like symptoms in wild rodents, similar to those in laboratory rats [35–37] or mice [38].

Our results corroborate previous results on possible PTSD-like symptoms in adult female wild captive wolves (*Canis lupus*), elephants, chimpanzees and birds (black-capped chickadees, *Poecile atricapillus*) [39–42].

### HPA-axis

Retrospective analysis of FCM concentrations collected 1–2 hours after predator exposure showed that among all wild rodent species, the PTSD phenotype individuals were typified by a blunting of the FCM response to the stressor, which was significantly different from those observed in less or non-affected groups. As expected, rodents with less extreme patterns of behavioral responses (partial or minimal behavioral disruption) displayed a significant increase in FCM stress response. The validity of these findings was supported by the results of the Pearson correlation analysis performed irrespective of the CBC classification, which revealed that FCM concentrations were significantly and negatively correlated with the anxiety index, indicating that lower FCM concentrations shortly after exposure predicted higher anxiety levels overall.

The blunted cortisol response indicates an underlying dysfunction in the overall dynamic modulation action of the HPA axis in rodents with the PTSD phenotype. This blunted FCM concentration could prolong the availability of norepinephrine to synapses in both the periphery and brain [43–45], which, in turn, might affect the consolidation of the memory of the traumatic event. Adrenergic activation in the face of low cortisol levels facilitates fear memory in animals [46]. Additionally, glucocorticoids play an important role during foraging, as their release controls appetite and food intake [47]. Allenby’s gerbils implanted with cortisol foraged longer, but harvested food more slowly due to greater vigilance and apprehension than placebo-treated gerbils [48]. The authors suggested that glucocorticoids affect energy acquisition and provide a physiological context to explain how foragers manage risks and the trade-off between food and safety [48]. Gutman et al. [34] studied FCM levels and foraging of nocturnal *A-Cahirinus* and diurnal *Acomys russatus* and found that both species exhibited high FCM concentrations and reduced foraging when the moon was full, suggesting that reduced foraging may be mediated by increased glucocorticoid concentrations [34]. Tree lizards treated with exogenous glucocorticoids responded more quickly to predator stress and hid for a longer duration (decreased feeding effort/reduced foraging) than did control lizards [49]. Moreover, the HPA axis of wild strain birds reacted more quickly to capture and handling (corticosterone levels increase within 250 s) and more strongly (higher corticosterone peak values) than the domesticated strain [50]. Together with our previous results and the ecology literature (which is not limited to laboratory rodents), a clearer picture of the HPA axis as a contributing factor to the development of PTSD is starting to emerge. Based on this evidence, we hypothesize that during an acute threat, the quick initiation and strong modulation action of the HPA axis should also entail rapid termination of the physiological stress response through negative feedback inhibition (short-term duration) once the treatment has passed [51]. Rapid return to the basal state renews foraging behavior via its effect on locomotor activity, which leads to better foraging capacity and increased dispersal [51–53]. Increased dispersal may be an optimal strategy to escape stressful small-scale events that persist over long periods of time [52]. All the factors mentioned above could lead to a return to routine, that is, faster recovery and preparedness to deal with subsequent stressors. Quick initiation accompanied by rapid termination of the HPA axis can directly or indirectly modify behavior and enhance individual survival. In contrast, in the absence of a rapid and strong HPA-axis response, rodents with the PTSD phenotype are at greater risk of predation. However, because predators represent an emergency that may require split-second responses for survival, a delayed, slower, or faulty release of glucocorticoid modulation action may result in costly or even fatal consequences.

In *G-Gerbillus*, we found significant intersexual differences in FCM concentrations, with females demonstrating significantly higher levels than males. This is interesting because, in this species, we also observed fewer PTSD phenotypes in females than in males. These findings imply that higher levels of cortisol could be protective against the development of PTSD and might explain the disparity in findings in rodent and human studies. Female mice and rats generally demonstrate higher plasma and adrenal corticosterone levels and more adaptive stress responses [54–60], whereas men (humans) have higher basal cortisol levels associated with a lower prevalence of stress-related psychopathology [61, 62]. Therefore, one might hypothesize that higher basal cortisol levels are protective against the development of PTSD.

As the HPA axis may serve as a key mechanism of development, modulating growth, and maintenance across a diverse array of taxa, a prolonged glucocorticoid stress response bears the risk of high stress-induced physiological and energetic costs (depletion of energetic stores) [50–52, 63], which could also impair reproduction.

In the wild, individual basal body condition, which is an important factor in determining survival, is also a major component of physiological stress responses [52]. A factor that is less considered in laboratory conditions (according to ethics instructions, individuals who lose weight or show signs of body neglect or illness are excluded from studies).

### Orexin-A levels

Next, we analyzed harvested brains to assess the consequences of predator stress on orexin-A levels in the hypothalamic nucleus. Orexin neurons, which are considered “multi-tasking” neurons, receive a variety of signals related to environmental, physiological, and emotional stimuli and project broadly to the entire central nervous system [64–67]. Orexin neurons orchestrate various aspects of survival via regulation of feeding behavior (energy homeostasis) and sleep-wakefulness [67]. We found that eight days after stress exposure, orexin-A levels in the hypothalamus were downregulated in animals whose behaviors were severely affected by the stressor (PTSD phenotype) in all three species but did not change in the PBR and MBR groups in all species. Moreover, there was a striking negative correlation between the severity of the anxiety index and changes in the hypothalamus orexin-A levels in all three species. Pearson’s correlation analysis revealed that the downregulation of orexin-A levels was significantly correlated with an increased degree of anxiety-related behaviors, assessed by EPM, across all samples in each species.

The reduction in hypothalamic orexin-A levels eight days after stress exposure in PTSD phenotype rodents may have profound ecological implications. The orexinergic system initiates, coordinates, and maintains survival behaviors and survival-related processes (unified orexinergic survival theory) [68]. When the orexinergic system is activated in response to stress or alerting stimuli, orexin neurons can initiate (and maintain) behavioral stress responses by activating arousal, sensory, somatomotor, visceromotor, hormonal, and other systems, enabling animals to better prepare for, respond to, and cope with the acute demands of physical and emotional threats to re-establish homeostasis [69]. In the wild, animals exhibit reactionary prey behaviors when orexinergic neurons in the lateral hypothalamus of prey animals are activated by adequate exteroceptive inputs detailing the presence of a hungry predator [68]. Accordingly, adequate survival responses require that animals maximize the functioning of their sensory systems to critically evaluate their environmental changes, such as threats or other life-threatening circumstances (i.e., food deprivation) [68]. Thus, the decreased levels of hypothalamic orexin-A in the PTSD phenotype eight days after predator stress across all three species may affect survival behaviors and survival-related processes, such as reduced alertness to potential threats, diminished ability to respond proportionally to threats, which together may increase predation risk.

These findings are supported by our previous report that the orexinergic system orchestrates various aspects of survival behaviors in response to predator stress and is related to the pathophysiology of PTSD [69]. Our findings are also supported by previous studies that investigated orexin expression in patients with PTSD [70]. It has been demonstrated that cerebrospinal fluid (CSF) and plasma orexin-A levels are significantly lower in patients with PTSD than in healthy controls, and CSF orexin-A levels are strongly and negatively correlated with PTSD severity, as measured by the Clinician-Administered PTSD Scale, in patients with PTSD [70].

### Morphological changes

The morphological characteristics of DG granule cells were evaluated using the Golgi-Cox method in all animals. All wild rodent species whose behavior was extremely disrupted (PTSD phenotype) selectively displayed significantly lower total dendritic length along the DG neurons. The implications of these results are that rodents with the PTSD phenotype were characterized by severe atrophy in the DG subregion. Since the dendritic arbor is responsible for receiving and consolidating neuronal information input [71–73], the reduced dendritic arbor in the DG in PTSD phenotype rodents can have considerable consequences for the functional properties of cells and neuronal circuitry, including decreased synaptic plasticity and synaptic strength and impaired stabilization of synaptic connectivity, which may in turn lead to vulnerability to psychopathology.

In contrast, rodents whose behavior was minimally affected or unaffected (MBR) displayed significantly longer total dendritic lengths and longer dendritic branches along the DG neurons. In all wild rodent species that displayed partial behavioral responses, the morphological response was intermediate or identical to that of the MBR group. These findings were consistent with our previous findings in rats [73]. Thus, these results suggest that predator cue exposure leads to dendritic atrophy of dentate granule cells, which probably decreases the amount of information that neurons can obtain from the environment.

One limitation of this study is the relatively small sample sizes employed. To establish the long-term, non-lethal effects of predators on wild rodents, further research incorporating larger sample sizes and longitudinal follow-up tests is necessary.

In summary, in wild rodent species, the prevalence of PTSD phenotype was found to be 21.7% to 26.7% of the total population, similar to that seen in laboratory rodents or individuals with a history of trauma. In all three rodent species, individuals who developed the PTSD phenotype to the predator stimuli were characterized by a blunting of the FCM response, a lower hypothalamic orexin-A level, and lower total dendritic length in the DG granule cells eight days after exposure. Phenotypically, this results in a significant functional impairment, potentially impacting reproduction and survival, mediating indirect effects of predators on prey demographics.

Allen et al. [74] experimentally manipulated fear in free-living wild songbird populations over three annual breeding seasons by intermittently broadcasting playbacks of either predator or non-predator vocalizations and comprehensively quantified the effects on all components of population growth, together with evidence of a transgenerational impact on offspring survival as adults. They found that fear itself significantly reduced the population growth rate by causing cumulative, compounding adverse effects on fecundity and every component of offspring survival, resulting in predator playback parents producing 53% fewer recruits to the adult breeding population [74].

## Conclusions

Although PTSD is defined in terms of human responses, several behavioral, physiological, and neurobiological survival responses are shared between humans and other mammals (i.e., learning and remembering about danger and responding to or avoiding situations that present life-threatening risks), suggesting that just as the adapted stress/threat response has evolutionary roots, the post-traumatic stress syndrome has evolutionary roots as well. Gerbils, wolves, elephants, and marine iguanas may seem evolutionarily far from humans, but there are compelling scientific reports to hypothesize that the ecology of fear [2], which may manifest differently across different taxa, may be largely the same.

Contrary to the hypothesis that the impact of a predator on an individual prey is either fatal or transitory (i.e., “fight or flight”), it seems that some individuals that survive predation stress do not continue their lives as before, but display long-lasting behavioral and morphological dysfunction, that resemble human PTSD responses, which could affect reproduction and survival. The finding that PTSD symptoms may affect 1 in every 5 wild rodents, which could negatively impact their fitness, has significant ramifications for study animal behavior and population dynamics.

### Supplementary information

SUPPLEMENTAL MATERIA
