# Supplementary material for: Sounds of danger and post-traumatic stress responses in wild rodents: ecological validity of a translational model of post-traumatic stress disorder
Source: Mol Psychiatry. 2023 Sep 6;28(11):4719–28. doi: 10.1038/s41380-023-02240-7 (PMC10914612; doi:10.1038/s41380-023-02240-7)
Supplement: Supplementary file 1 — SUPPLEMENTAL MATERIA [file 41380_2023_2240_MOESM1_ESM.docx]

**Supplementary Materials #1**

The spiny mouse is a nocturnal rodent species native to regions of Africa, the Middle East, and Europe, where it inhabits sandy deserts and rocky terrain (1). They have a relatively long gestation period (39–40 days), a small litter size (1–4, usually 1–3), and precocial pups. Importantly, the spiny mouse adrenal gland is similar to the human adrenal gland, as it secretes cortisol as the major circulating glucocorticoid and can also synthesize and secrete dehydroepiandrosterone (2), an important androgen involved in brain development that conventional mice and rats do not produce in utero. The common spiny mouse differs from laboratory rats (*Rattus norvegicus*) and mice (*Mus musculus*) in many aspects of exploratory behavior, such as in-depth perception, distance perception, exploration, and excitability, which are important for both estimating danger and responses in a changing environment and prey pressure (3, 4). These differences are paralleled by differences in brain anatomy (5).

**Supplementary Materials #2**

**Classification according to Cut-Off Behavioral Criteria (CBC)**

Human responses to traumatic experiences clearly vary greatly in extent and in character. More importantly, PTSD occurs in a percentage (roughly 25-35%) of those exposed, underscoring the importance of contemporary definitions of stress-related disorders (DSM and ICD) and of inclusion and exclusion criteria applied in controlled clinical trials. In contrast, most animal studies have tended to relate to unclassified “global” groups, i.e., the entire exposed population versus control populations without distinction, whereas researchers who work with animals have long been aware that individual study subjects tend to display a varying range of responses to stimuli, certainly where stress paradigms are concerned. The heterogeneity in animal responses might be regarded as confirming the validity of animal studies, rather than as a problem. It stands to reason that a model of diagnostic criteria for psychiatric disorders could be applied to animal responses to augment the validity of study data, as long as the criteria for classification are clearly defined, reliably reproducible and yield results that conform to findings in human subjects.

In order to model DSM-4 criteria for PTSD, we developed “the Cut-off Behavioral criteria” model of PTSD (6-10). The behavioral responses of animals in both the elevated plus maze and acoustic startle response tests were first analyzed by group. Subsequently, individual animals were classified according to their behavioral response pattern on both the elevated plus maze and acoustic startle response, by using the cut-off behavioral criteria model, as exhibiting either “extreme behavioral response” (EBR) or “minimal behavioral response” (MBR) (9, 10). Those that fulfilled neither set of criteria were labeled, exhibiting a “partial behavioral response” (PBR). This procedure is detailed in Figure S1.

**
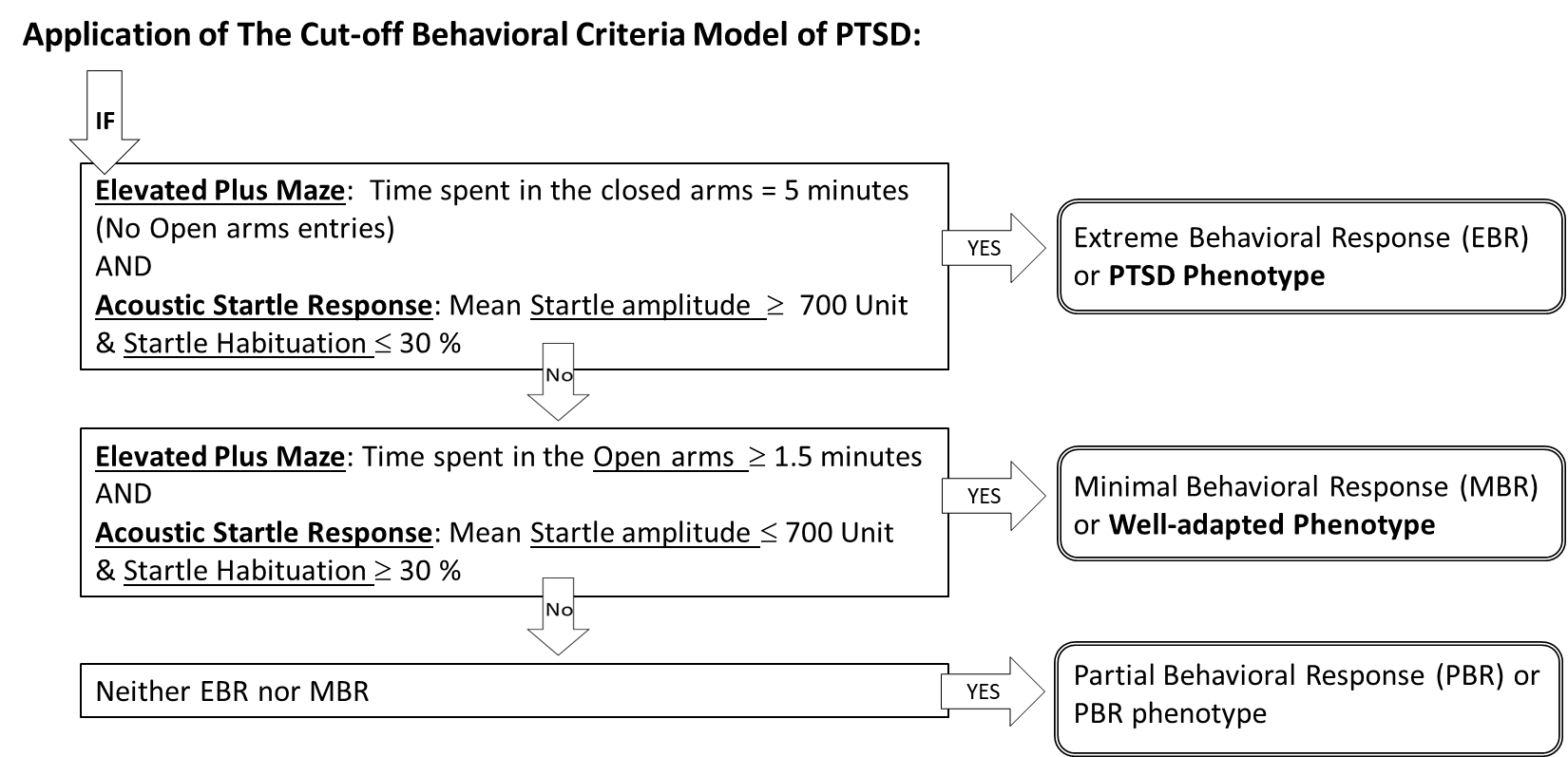
**

**Figure S1.** The cut-off behavioral criteria algorithm: To approximate the behavioral model to contemporary clinical conceptions of post-traumatic stress disorder (PTSD), we classified animals into groups according to the degree of response to the stressor (PSS, predator scent stressor), i.e., the degree to which the behavior of an individual is altered or disrupted. To this end, behavioral criteria were defined and then complemented by the definition of cut-off criteria, which reflect the severity of response; this parallels inclusion and exclusion criteria applied in clinical research. The procedure requires the following steps: (**A**) Verification of global effect: the data must demonstrate that the stressor has a significant effect on the overall behavior of PSS- versus Sham PSS-exposed populations at the time of assessment; (**B**) Application of the cut-off behavioral criteria to the data: to maximize the resolution and minimize false positives, extreme responses to the stress in both the elevated plus-maze and acoustic startle response paradigms (performed sequentially) are required for “inclusion” into the extreme behavioral response (EBR) group. A negligible response in both paradigms is required for inclusion into the minimal behavioral response (MBR) group. Individuals that are not classified as having an EBR or an MBR are, by default, classified as having a partial behavioral response (PBR).

**Supplementary Materials #3**

**3:1. Behavioral assessments**

All experimental protocols met the requirements of the 1994 Law for the Prevention of Cruelty to Animals (Experiments on Animals) of the State of Israel and were approved by the Ben Gurion University Committee for the Ethical Care and Use of Animals in Experiments.

**Owl territorial calls** (playback of tape-recording) were chosen as stressors for this study. In the predator model of PTSD, the extremity of the stressors faced by animals in the wild, in a real-world context, would appear to emulate the circumstances leading to PTSD in humans more closely. In general, owl territorial calls have been demonstrated to be effective in various rodents, including spiny mice and gerbils (11, 12).

All behavioral tests were performed in a closed, quiet, light-controlled room in the Faculty of Medicine, Anxiety and Stress Research Unit, Ben-Gurion University between 10:00 and 14:00 h. All behavioral tests were video-recorded for future analysis using the ETHO-VISION program (Noldus), by an investigator blinded to the experimental protocol. All experiments and measurements handled by an experimenter blinded to groups conditions.

**Open field test:** To assess spontaneous activity levels, each animal was tested in a large open arena composed of a 50 × 50 cm square box with uniformly black walls 60 cm high under white light of 2.8 lux (13). Movement was recorded using a video camera for 10 min. The parameters assessed were the time and distance moved in the inner, middle, and outer zones. Time spent in the inner zone, in the middle zone, and along the perimeter were calculated as a percentage of the total time, in order to show how long the rodent stayed in the vicinity of the walls of the arena, compared with the time spent in the center of the arena or in the middle zone. The open field apparatus was cleaned after each session using 70% ethyl alcohol and allowed to dry between tests.

**Elevated plus maze:** The EPM is frequently used to evaluate anxiety-like behavior in rodents (14). The EPM consists of a plus-shaped platform with two open arms and two closed arms, surrounded by 15 cm high opaque walls on three sides, with arms of the same type located opposite each other. The plus maze was elevated 50 cm above the floor, and light levels in the open arms were 200 lux.

Each rodent was placed on a central platform facing an open arm and allowed to explore the maze for 5 min. An independent observer videotaped and recorded each test. Arm entry was defined as entering the arm with all four paws. At the end of each session, the animals were returned to their cages, and the maze was cleaned with 70% ethanol before the start of the next session. The following parameters were measured: duration in open and closed arms and on the central platform, open and closed arm entries, and total entries into all arms (total exploration). “Anxiety Index,” an index that integrates the EPM behavioral measures, was calculated as follows:

$$Anxiety Index=1-\left[ \frac{\left( \frac{time spent in the open arms}{total time on the maze} \right)+\left( \frac{number of enteries to the open arms}{total exploration on the maze} \right)}{2} \right]$$

Anxiety Index values range from 0–1 where an increase in the index indicates increased anxiety-like behavior.

**Acoustic startle response:** The startle response was measured using two ventilated startle chambers (SR-LAB system, San Diego Instruments, San Diego, CA, USA). The SR-LAB calibration unit was used routinely to ensure consistent stabilimeter sensitivity between the test chambers over time. Each Plexiglas cylinder rested on a platform inside a soundproofed, ventilated chamber. Movement inside the tube was detected using a piezoelectric accelerometer placed below the frame. The sound levels within each test chamber were measured routinely using a sound level meter (Radio Shack) to ensure consistent presentation. Each test session started with a 5-min acclimatization period to background white noise of 68 dB, followed by 30 acoustic startle trial stimuli in six blocks (110 dB white noise of 40 ms duration with 30 or 45 s inter-trial intervals). Behavioral assessment consisted of the mean startle amplitude (averaged over all 30 trials) and the percentage of startle habituation to repeated presentation of the acoustic pulse. Percent habituation, the percent change between the responses to the first and last (6th) blocks of sound stimuli was calculated as follows:

$$Percent Habituation= 100 \times\left[ \frac{\left( average startle amplitude in Block 1 \right)+\left( average startle amplitude in Block 6 \right)}{average startle amplitude in Block 1} \right]$$

The experiment was run in a number of trials because we tested mixed species groups (with each animal in its own cage) of 10–12 animals at a time.

**3:2. Golgi-Cox Staining**

To obtain accurate measurements of dendritic parameters, strict criteria were adopted for the selection of filled neurons before quantitative analysis: 1) only well-impregnated neurons were chosen for histological analysis; 2) granule cells were included in this analysis only if the cell bodies and primary dendrites were clearly stained and easily distinguishable from the neighboring cell bodies and their dendrites; 3) granule cells were sampled from the suprapyramidal blades of the dentate gyrus (DG) in both the right and left sides of the brain; 4) granule cells from the inner granule zone (IGZ) were included in this analysis (because the dendritic morphology of hippocampal DG cells varies with their position in the granule cell layer (15)). A cell was classified as belonging to the IGZ if the entire soma was positioned in the inner half of the granule cells layer (GCL). Granule cells whose soma intersected the midline of the GCL, the outer granule zone (OGZ), or the subgranular zone were not included in any analyses.

We performed an analysis to characterize the extent to which dendrites branched out from both somatic and dendritic sites. Primary dendrites were defined as direct extensions from a soma at least 10 µm in length. Only DGs with at least one primary dendrite > 10 µm in total length were analyzed. When a primary dendrite bifurcated at a branch point, the dendrites extending from that branch point were classified as secondary. We extended this analysis to include tertiary-(3), quaternary-(4), quinary-(5), and senary-order (6) dendrites. This procedure provides an additional measure of the dendritic arborization pattern, allowing a more comprehensive analysis of the differences in the dendritic branch patterns. We also performed Sholl analysis (16). A series of concentric rings, spaced 25 μm apart, were placed over the neuron and centered on the cell body, and the number of dendrite crossings as a function of distance was recorded.

All slides were coded, and the analysis was performed by an experimenter blinded to the origin of the slides. The dendritic morphology was observed using epifluorescence microscopy (Leica, Germany). A 0.5 µm interval *z*-series was captured throughout the extent of the dendritic arbor of the DG with a CCD camera (Leica, Germany) controlled by LAS software.

**Supplementary Materials #4**

**4:0: RESULTS:** Open field paradigm

To preclude the possible effects of basal pre-trauma anxiety, which can be a risk factor for the development and persistence of PTSD, all animals were first evaluated in the open field paradigm under basal conditions. The open-field assay simultaneously measures locomotion and anxiety in rodents by quantifying distance traveled and affinity toward the periphery of the testing arena (i.e., thigmotaxis).

| **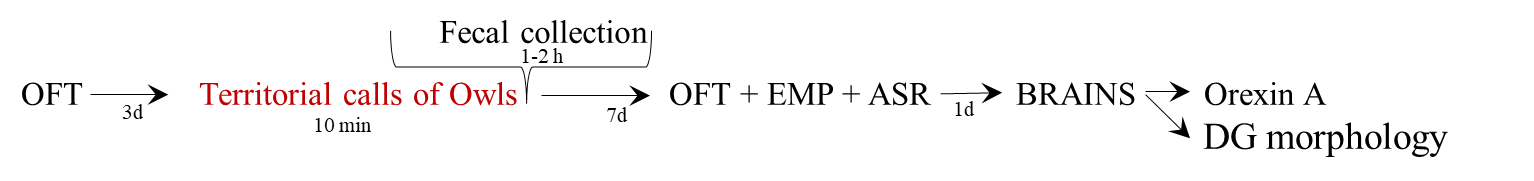** | | |
| --- | --- | --- |
| a | b | c |
| **** | **** | **** |
| d | | |
| 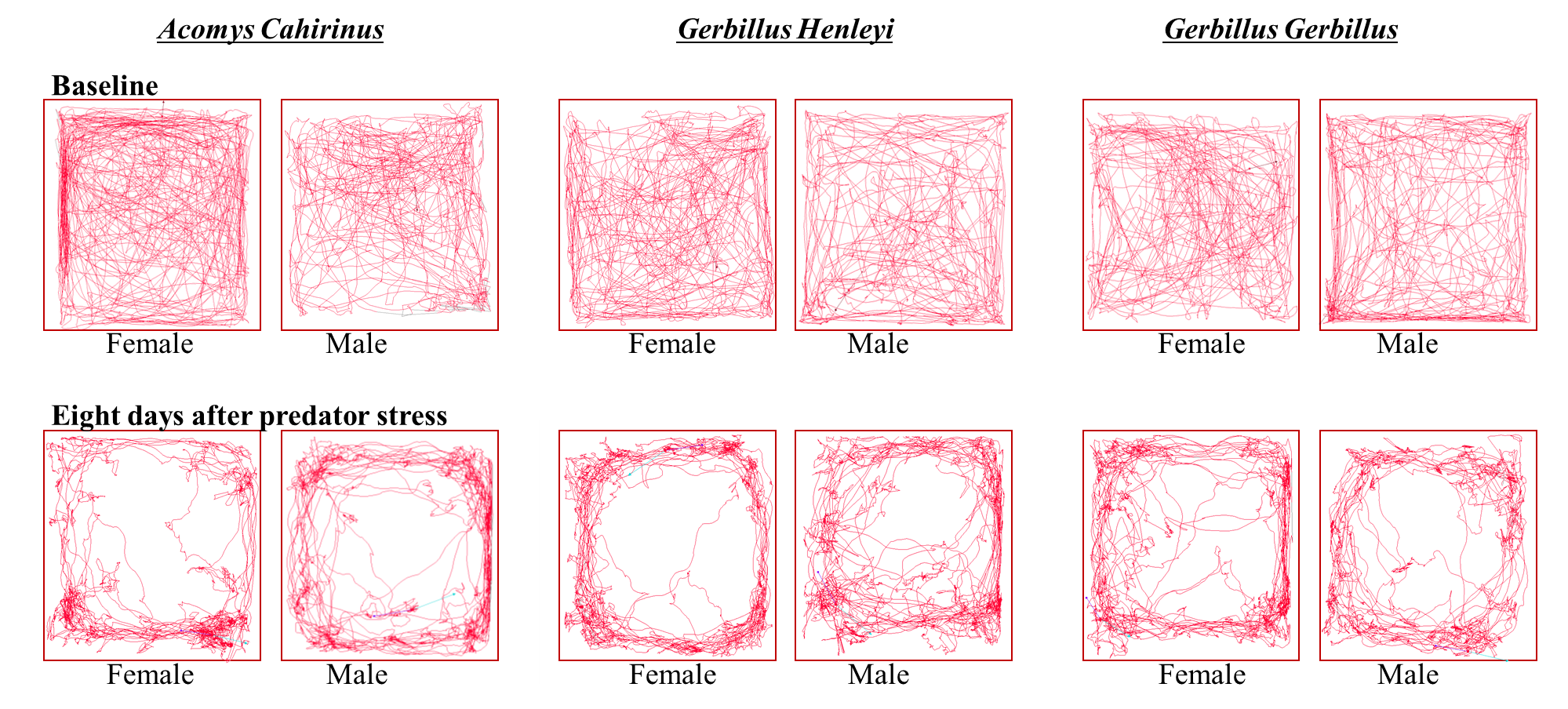 | | |
| **Figure S3. Open Field spontaneous activity during basal conditions and seven days after predator cue exposure:** The top panel depicts the experimental protocol. The red circle signifies the behavioral test performed, for which the results are shown. (A) Percentage of time spent in the inner zone of the arena, (B) percentage of time spent in the middle zone of the arena, and (C) percentage of time spent in the outer zone of the arena. (D) Representative patterns of locomotor activity (cumulative values) in the arena in all species (female and male) under basal conditions (before predator stress). (E) Representative patterns of locomotor activity (cumulative values) in the arena in all species (female and male) seven days after stress exposure. Under basal conditions, the level of anxiety-related behavior did not differ between species and sexes. All species reacted to the owl vocalizations by reducing the percentage of time spent in the inner and middle zones and increasing the time spent in the outer zone, which is a common index of anxiety-related behavior. Bars represent group mean ± SEM. | | |

Under baseline conditions, no differences were found between species, sex or species-sex interaction in the time spent in the inner, middle, or outer (the thigmotaxis area, i.e., the tendency to remain close to vertical surfaces) zones of the arena (Figure S2a-d). Two-way ANOVA observed no effects for species, sex, or species-sex interaction. All species possessed an inherent curiosity of a novel environment and spent more than 50% of their time along the perimeter zone, the rest of the time mostly in the middle zone (approximately 30%) and in the inner zone (approximately 15%). All species moved through the center in a convoluted path, frequently changing the direction of progression. No significant differences were observed in the velocity and distance moved between the species and sexes. The results obtained demonstrated that during baseline conditions, the level of anxiety-related behavior was low: the locomotion patterns, the traveled distances, and the mean time spent in the inner, middle, or outer arena zone all indicated a low level of anxiety-related behavior without any differences among the species or between sexes.

8 days after predator exposure: In the inner, middle, and outer arena zones, two-way RM-ANOVA revealed a significant effect of stress (F(1,67)=197.5, p<0.0001, F(1,67)=445, p<0.0001, and F(1,67)=528, p<0.0001, respectively). No effects were observed for species, sex, species-sex, stress-species, stress-sex, or stress-species-sex interactions. In all species, stress reduced the percentage of time spent in the inner and middle zones and increased the time spent in the outer zone, which are common indices of anxiety-related behavior. This response pattern was not significantly different among species or between sexes.

**4:1. RESULTS: elevated plus mase**

| 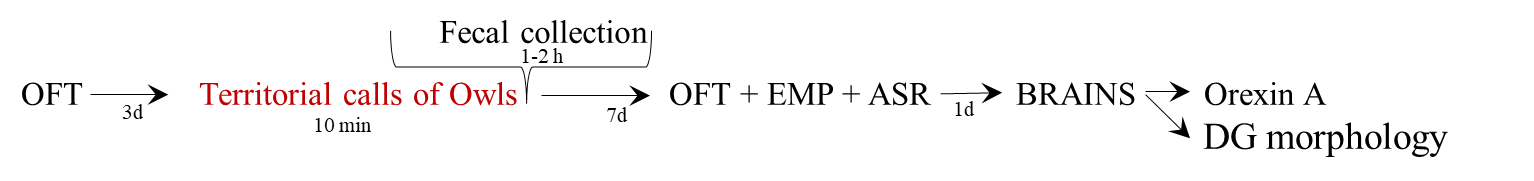 | | | | |
| --- | --- | --- | --- | --- |
| a | | b | | c |
|  |  | |  | |
| d | e | | f | |
|  |  | |  | |
| **Figure S4. The long-term effects of predator cue exposure on behavior:** The top panel (1) depicts the experimental protocol. The red circle signifies the behavioral test performed, for which the results are shown. (a) Time (min) spent in the open arms of the elevated plus maze (EPM). (b) Time (min) spent in the closed arms of the elevated plus maze. (c) Time (min) spent in the central platform of the elevated plus maze. (d) Entries to the open arms of the maze. (E) Entries to the closed arms of the maze.  (f) Total arm entries (total exploration/activity). Owl territory calls had long-lasting influences on rodent behavior; all species reacted significantly to predator cue stress in terms of anxiety-related behavior in the EPM and ASR paradigms 7 days after exposure. Data are presented as data points and mean ± SEM. | | | | |

In terms of time spent in the open arms (Figure S4a) and time spent in the closed arms (Figure S4b), two-way ANOVA revealed a significant effect of species (F(2,67)=8.6, P<0.0005 and F(2,67)=5.2, P<0.008, respectively) and sex (F(2,67)=6.85, p<0.015 and F(2,67)=6.4, P<0.015, respectively). No effects were observed for species-sex interactions. Post-hoc Bonferroni test confirmed that female *Acomys cahirinus* exposed to predator stress spent significantly less time in the open arms and entered the open arms less frequently than female *Gerbillus gerbillus* (p<0.005 and p<00002, respectively). In terms of open-arm entries (Figure S4d) and total activity (Figure S4f), there were significant effects of species (F(2,67)=15.4, P<0.0001 and F(2,67)=4.2, P<0.025, respectively). No effects were observed for sex or species-sex interactions. Post-hoc Bonferroni test confirmed that male *Acomys cahirinus* exposed to predator stress entered the open arms less frequently than female *Gerbillus gerbillus* (p<0.04). No species or sex differences in time spent in the central platform of the maze (Figure 2C) were observed.

**REFERENCES**

1. Ratnayake U, Quinn T, Daruwalla K, Dickinson H, Walker DW (2014): Understanding the behavioural phenotype of the precocial spiny mouse. *Behavioural brain research*. 275:62-71.

2. Quinn TA, Ratnayake U, Dickinson H, Nguyen TH, McIntosh M, Castillo-Melendez M, et al. (2013): Ontogeny of the adrenal gland in the spiny mouse, with particular reference to production of the steroids cortisol and dehydroepiandrosterone. *Endocrinology*. 154:1190-1201.

3. Brunjes P (1990): The precocial mouse, Acomys cahirinus. *Psychobiol*. 18:339-350.

4. Eilam D (2004): Locomotor activity in common spiny mice (Acomys cahirinuse): the effect of light and environmental complexity. *BMC ecology*. 4:16.

5. D'Udine B, Alleva E (1988): The Acomys cahirinus (spiny mouse) as a new model for biological and neurobehavioural studies. *Polish journal of pharmacology and pharmacy*. 40:525-534.

6. Cohen H, Matar MA, Joseph Z (2013): Animal models of post-traumatic stress disorder. *Curr Protoc Neurosci*. Chapter 9:Unit9 45.

7. Cohen H, Zohar J (2004): An animal model of posttraumatic stress disorder: the use of cut-off behavioral criteria. *Ann N Y Acad Sci*. 1032:167-178.

8. Cohen H, Zohar J, Gidron Y, Matar MA, Belkind D, Loewenthal U, et al. (2006): Blunted HPA axis response to stress influences susceptibility to posttraumatic stress response in rats. *Biol Psychiatry*. 59:1208-1218.

9. Cohen H, Zohar J, Matar M (2003): The relevance of differential response to trauma in an animal model of posttraumatic stress disorder. *Biol Psychiatry*. 53:463-473.

10. Cohen H, Zohar J, Matar MA, Kaplan Z, Geva AB (2005): Unsupervised fuzzy clustering analysis supports behavioral cutoff criteria in an animal model of posttraumatic stress disorder. *Biol Psychiatry*. 58:640-650.

11. Eilam D, Dayan T, Ben-Eliyahu S, Schulman II, Shefer G, Hendrie CA (1999): Differential behavioural and hormonal responses of voles and spiny mice to owl calls. *Animal behaviour*. 58:1085-1093.

12. Gutman R, Dayan T, Levy O, Schubert I, Kronfeld-Schor N (2011): The effect of the lunar cycle on fecal cortisol metabolite levels and foraging ecology of nocturnally and diurnally active spiny mice. *PloS one*. 6:e23446.

13. Walsh RN, Cummins RA (1976): The Open-Field Test: a critical review. *Psychological bulletin*. 83:482-504.

14. File SE (1993): The interplay of learning and anxiety in the elevated plus-maze. *Behavioural brain research*. 58:199-202.

15. Green EJ, Juraska JM (1985): The dendritic morphology of hippocampal dentate granule cells varies with their position in the granule cell layer: a quantitative Golgi study. *Exp Brain Res*. 59:582-586.

16. Sholl DA (1956): The measurable parameters of the cerebral cortex and their significance in its organization. *Prog Neurobiol* 2:324-333.
